# Comparison of whole-body 18F-FDG PET/CT and PET/MRI for distant metastases in patients with malignant tumors: a meta-analysis

**DOI:** 10.1186/s12885-022-10493-8

**Published:** 2023-01-10

**Authors:** Cici Zhang, Zhishan Liang, Wei Liu, Xuwen Zeng, Yuzhen Mo

**Affiliations:** 1 Department of Radiology, Guangzhou Red Cross Hospital, Guangzhou, China; 2 Department of Cardiology, The People’s Hospital of Guangxi Zhuang Autonomous Region, Nanning, China; 3 Department of Breast, Guangzhou Red Cross Hospital, Guangzhou, China; 4 Department of Radiotherapy, Guangzhou Red Cross Hospital, No.396, TongFu Road, HaiZhu District, Guangzhou, 510220 Guangdong China

**Keywords:** Tumor, Distant metastases, Positron emission tomography/magnetic resonance imaging, Positron emission tomography/computed tomography

## Abstract

**Background:**

As a first-line imaging modality, whole-body fluorine-18 fluorodeoxyglucose (18F-FDG) positron emission tomography (PET)/computed tomography (CT) and 18F-FDG PET/magnetic resonance imaging (MRI) had been widely applied in clinical practice. However, 18F-FDG PET/MRI may be superior to PET/CT for the diagnosis of distant metastases in patients with advanced-stage. Therefore, it is timely and important to systematically determine the diagnostic accuracy of 18F-FDG PET/MRI compared with that of 18F-FDG PET/CT for the diagnosis of distant metastases.

**Methods:**

This study aimed to compare the diagnostic accuracy of 18F-FDG PET/CT and PET/MRI for the diagnosis of distant metastases in patients with malignant tumors. Relevant studies using both 18F-FDG PET/CT and PET/MRI for assessment of distant metastases in patients with malignant tumors were searched in PubMed, Embase, The Cochrane Library, and Scopus from January 2010 to November 2023. Two reviewers independently selected studies according to the inclusion and exclusion criteria. A reviewer extracted relevant data and assessed the quality of the eligible studies. The pooled sensitivity, specificity, positive likelihood ratio, negative likelihood ratio, and area under the summary receiver operating characteristic curve (AUC) for 18F-FDG PET/CT and PET/MRI were analyzed. Subgroup analysis was performed.

**Results:**

Across 14 studies (1042 patients), 18F-FDG PET/MRI had a higher sensitivity (0.87 versus 0.81), AUC value (0.98 versus 0.95), and similar specificity (0.97 versus 0.97), than PET/CT for detecting distant metastases. In 3 studies of breast cancer (182 patients), 18F-FDG PET/MRI had a higher sensitivity (0.95 versus 0.87) and specificity (0.96 versus 0.94) than PET/CT. In 5 studies of lung cancer (429 patients), 18F-FDG PET/CT had a higher sensitivity (0.87 versus 0.84) and a lower specificity (0.95 versus 0.96) to PET/MRI.

**Conclusions:**

18F-FDG PET/MRI and PET/CT both performed well as detectors of distant metastases in patients with malignant tumors, and the former has higher sensitivity. The subgroup analysis highlights that 18F-FDG PET/MRI and PET/CT hold different advantages for distant metastases staging in different tumors, PET/MRI has a higher accuracy in patients with breast cancer patients, while PET/CT has a higher accuracy in patients with lung cancer.

## Background

Malignant tumors are common public health problems worldwide and lead to tumor-related complications and death. The presence of distant metastases is an important prognostic factor in patients with advanced malignant tumors. Accurate distant metastases staging is a critical initial step in choosing an appropriate therapeutic plan and predicting patient prognosis.

At present, available whole-body tumor staging tools clinically include computed tomography (CT), magnetic resonance imaging (MRI), fluorine-18 fluorodeoxyglucose (18F-FDG) positron emission tomography (PET)/CT, and PET/MRI. Although PET/CT is the conventional imaging procedure used to depict malignant lesions and perform tumor staging because of its high speed, high diagnostic accuracy, and availability, however, it has several limitations, such as ionizing radiation and the inability to detect sub-centimeter lesions in the liver and brain [1]. In contrast, PET/MRI has the advantage of combining the metabolic information provided by PET and the unique features of MRI, including avoidance of radiation exposure and high soft tissue contrast; therefore, PET/MRI is rapidly emerging as an important imaging modality for assessing tumor staging.

Several studies have reported on the diagnostic accuracy of 18F-FDG PET/MRI and PET/CT as promising imaging methods for the distant metastases staging, and the reported accuracies are variable, with a sensitivity ranging from 44 to 100% and a specificity ranging from 81 to 100% for PET/MRI, and with a sensitivity ranging from 44 to 100% and a specificity ranging from 75 to 100% for PET/CT [2–15], that limited to oncologic management decisions. Furthermore, as some studies included only a small number of subjects, the power of individual studies is limited. In addition, the most common sites for distant metastases are the lung, liver, brain, and bone, 18F-FDG PET/MRI shows superiority over PET/CT in detecting liver, brain, and bone metastases, due to dynamic contrast-enhanced imaging, diffusion-weighted imaging (DWI), and signal intensity (SI) assessment [5, 10, 14]. Therefore, 18F-FDG PET/MRI may be superior to PET/CT for the diagnosis of distant metastases. It is timely and important to systematically determine the diagnostic accuracy of distant metastases of 18F-FDG PET/MRI and compare it with that of 18F-FDG PET/CT.

## Materials and methods

### Literature search strategy

A combination of subject terms and free-text terms was mainly used to search the databases. The English terms 18F-FDG positron emission tomography/magnetic resonance imaging OR 18F-FDG PET/MRI AND positron emission tomography/computed tomography OR PET/CT AND distant metastases OR TNM staging AND cancer or tumor were searched in PubMed, Embase, The Cochrane Library, and Scopus from January 2010 to November 2023. No language restrictions on relevant studies during searching and selecting. To maximize the search results, the references of the retrieved articles were screened to identify additional studies.

The inclusion criteria for selecting studies were as follows: ① Whole-body 18F-FDG PET/MRI and PET/CT were both used to diagnose distant metastases in patients with malignant tumors regardless of the type of primary tumor. ② The primary tumor was confirmed by pathological analysis (biopsy or surgical specimens), and distant metastases were confirmed by pathological analysis (biopsy or surgical specimens) and/or imaging follow-up data (interval growth or stability). ③ The studies were based on a per-patient analysis. ④ The studies included greater than 10 patients. And the exclusion criteria were as follows: ① Non-original articles, such as conference abstracts, comments, letters to the editors, and reviews, were excluded. ② Studies in which diagnostic data could not be obtained were excluded. ③ Studies with data on only a per-lesion analysis were excluded.

### Literature selection and data extraction

In accordance with the inclusion and exclusion criteria, two reviewers (W Liu and ZS Liang) read the titles and abstracts of the literature independently, excluded articles that failed to meet the criteria, and the rest conducted full-text reading and data extraction those that met the criteria. The discussion was adopted in the case of disagreements. Data extraction included the following: ① study design (prospective or retrospective); ② general data (year of publication, authors); ③ basic features (number of eligible patients, the age range of eligible patients, locations of the primary tumor, data type (patient-based or lesion-based), reference standard, follow-up time, etc.); ④ technical characteristics of PET/MRI and PET/CT; and ⑤ outcome measures: true positives (TPs), true negatives (TNs), false positives (FPs), false negatives (FNs). For some studies that did not provide direct data, sensitivity, specificity, and accuracy were used to estimate the TPs, TNs, FPs, and FNs. When key information was lacking, the authors of eligible studies were contacted to supplement raw data.

### Risk of bias assessment

The updated Quality Assessment of Diagnostic Accuracy Studies (QUADAS-2) tool [16] was used by two independent researchers to perform a quality assessment of the studies. This updated tool allows for a more transparent rating of bias and applicability to diagnostic accuracy studies for four key domains (patient selection, index test, reference standard, and flow and timing). Each key domain was assessed as low risk, high risk, or unclear (inadequate information was provided).

### Statistical analysis

Diagnostic parameters were estimated based on patient data. Stata version 17 (Stata Corporation, TX, USA) was used to perform statistical analyses. Based on the data extracted from each study, we calculate the pooled sensitivity, specificity, positive likelihood ratio (PLR), negative likelihood ratio (NLR) of 18F-FDG PET/MRI and PET/CT for assessing distant metastases in patients with malignant tumors. We also calculated the area under the summary receiver operating characteristic curve (AUC). The AUC value for a perfect test is close to 1, while the AUC value for a poor test is close to 0.5.

The inconsistency index (*I*^*2*^) was used to evaluate the presence of heterogeneity between studies. If *I*^*2*^ < 50%, indicating that the heterogeneity was low, a fixed effects model was used, and if *I*^*2*^ > 50%, indicating that the heterogeneity was high and a random effects model was used. The presence of a threshold effect was analyzed by calculating the Spearman correlation coefficient, *P* < 0.05 was considered to indicate a threshold effect. When substantial heterogeneity was noted, subgroup analysis was performed.

#### Analysis of publication bias

A Deeks’ funnel plot was visually evaluated to determine any publication bias, with its statistical significance being examined using Deeks’ asymmetry test. A value of *P* < 0.05 was considered statistically significant.

## Results

### Process and results of literature selection

**Fig. 1:**
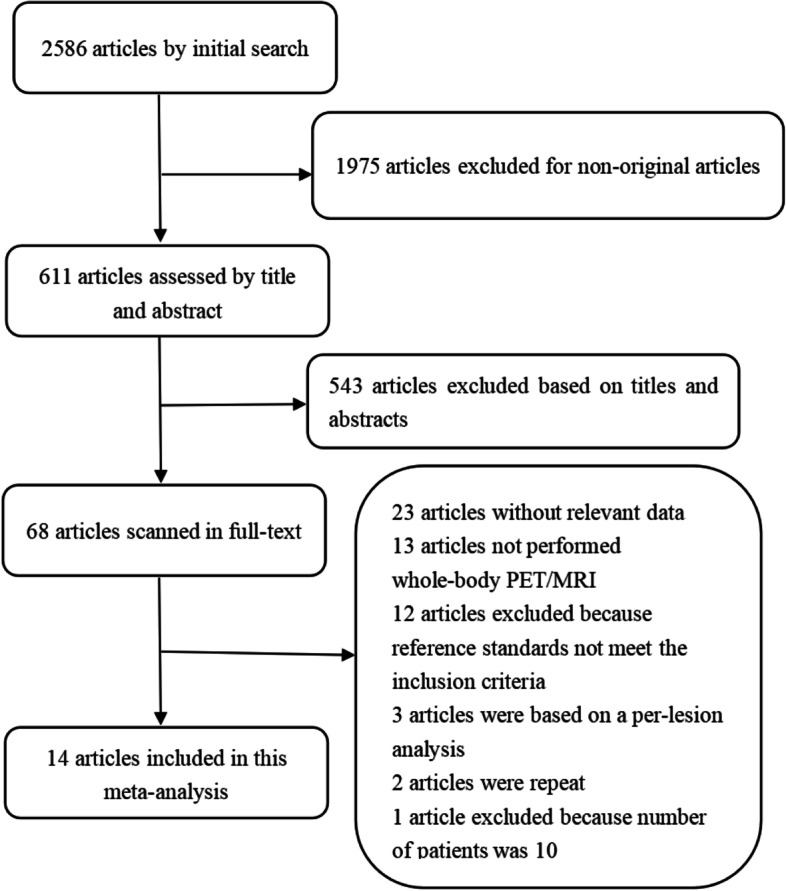
The flow diagram of literature search for the meta-analysis

The electronic search yielded 2568 articles, 1975 articles excluded for non-original articles, and 543 articles excluded based on titles and abstracts, the rest of the 68 articles were scanned in full-text and rejected 54 articles, a total of 14 articles (1042 patients) were finally eligible for meta-analysis. The flow diagram presenting the search history is shown in Fig. 1.

### Basic features of the included studies

Of 14 studies, 2 studies were retrospective, and 12 studies were prospective. In 5 studies of lung cancer were enrolled. In 3 studies of breast cancer were included. In 2 studies of various cancer patients were enrolled. In 2 studies of malignant pleural mesothelioma were enrolled. In 1 study of gastric cancer was included. In 1 study of pharynx squamous cell carcinoma was included. Table 1 displays the basic features of the included studies, and Table 2 shows the outcome measures of the included studies.

**Table 1:** Main characteristics of the studies included in the meta-analysis

Study	Year	Study design	N	Age	Cancer type	Data type	Reference standard	Follow-up time	PET/MRI	PET/CT
Ohno Y [2]	2015	Prosp	140	47–83	Lung cancer	Patient-based	Pathology and imaging follow-up	≥ 6 months	3.0 T -shiba Sequential CE: yes	Dose: 3.3 MBq/Kg Uptake time: 60 min
Heusch P [2]	2015	Retro	73	21–85	Various cancers	Patient-based	Pathology and imaging follow-up	273 days (median)	3.0 T Siemens Simultaneous CE: yes	Dose: 295 MBq(mean) Uptake time: 141 min(mean)
Lee SM [4]	2016	Prosp	45	35–79	Lung cancer	Patient-based	Pathology and imaging follow-up	393 days (median)	3.0 T Siemens Simultaneous CE: yes	Dose: 5.2 MBq/Kg Uptake time: 60 min
Melsaether AN [5]	2016	Prosp	51	32–76	Breast cancer	Patient & lesion-based	Pathology and imaging follow-up	≥ 6 months	3.0 T Siemens Simultaneous CE: yes	Dose: 547.6 MBq (mean) Uptake time:131 min
Huellner MW [6]	2016	Prosp	42	35–89	Lung cancer	Patient-based	Pathology and imaging follow-up	568 days (mean)	3.0 T GE Sequential CE: none	Dose: 350 MBq Uptake time: 60 min
Sekine T [7]	2017	Prosp	43	20–86	Occult tumors	Patient-based	Pathology and imaging follow-up	523–1848 days	3.0 T GE Sequential CE: 19 patients	Dose: 2 MBq/Kg Uptake time: 69 min(mean)
Catalano OA [8]	2017	Retro	51	20–71	Breast cancer	Patient-based	Pathology and imaging follow-up	≥ 24 months	3.0 T Siemens Simultaneous CE: yes	Dose: 4.44 MBq/kg Uptake time:60 min
Ohno Y [9]	2017	Prosp	64	56(mean)	Thymic epithelial tumor	Patient-based	Pathology and imaging follow-up	≥ 6 months	3.0 T Canon Sequential CE: yes	Dose: 132–300 MBq Uptake time:60 min
Botsikas D [10]	2019	Prosp	80	48(mean)	Breast cancer	Patient & lesion-based	Pathology and imaging follow-up	≥ 12 months	3.0 T Philips Sequential CE: yes	Dose: 3.5 MBq/kg Uptake time: 60 min
Ohno Y [11]	2019	Prosp	23	55–75	Malignant pleural mesothelioma	Patient-based	Pathology and imaging follow-up	6 or 12 months	3.0 T Canon Sequential CE: yes	Dose: 132–300 MBq Uptake time:60 min
Liu Y [12]	2019	Prosp	30	≥ 18	Gastric Cancer	Patient-based	Pathology and imaging follow-up	3.2–40.7 months	3.0 T Siemens Simultaneous CE: none	Dose: 2.22—4.44 MBq /kg Uptake time: 20–30 min
Yeh CH [13]	2020	Prosp	198	56(mean)	Pharynx squamous cell carcilowma	Patient & lesion-based	Pathology and imaging follow-up	≥ 12 months	3.0 T Siemens Simultaneous CE: yes	NR
Ohno Y [14]	2020	Prosp	104	43–85	Lung cancer	Patient-based	Pathology and imaging follow-up	≥ 24 months	3.0 T canon, 1.5 T Philips Sequential CE: yes	Dose: 3.3 MBq/kg Uptake time: 60 min
Ohno Y [15]	2021	Prosp	98	47–83	Lung cancer	Patient-based	Pathology and imaging follow-up	≥ 6 months	3.0 T Vantage Titan and Galan Sequential CE: yes	NR

*Abbreviations*: *N* Nunmber, *Prosp* Prospective, *Retro* Retrospective, *CE* Contrast enhanced, *NR* Not reported

**Table 2:** Outcomes measure of included studies

Study,year	PET/MR				PET/CT			
	TP	TN	FP	FN	TP	TN	FP	FN
Ohno Y [2], 2015	115	13	3	9	115	12	4	9
Heusch P [3], 2015	4	30	3	5	4	27	6	5
Lee SM [4], 2016	5	39	0	1	4	39	0	2
Melsaether AN [5], 2016	30	18	3	0	28	17	5	1
Huellner MW [6], 2016	11	26	4	1	12	28	2	0
Sekine T [7], 2017	11	31	1	0	9	32	0	2
Catalano OA [8] 2017	20	30	0	1	17	29	2	3
Ohno Y [9] 2017	4	60	0	0	2	60	0	2
Botsikas D [10] 2019	11	65	2	2	9	67	0	4
Ohno Y [11] 2019	2	20	0	1	3	19	1	0
Liu Y [12] 2019	3	22	0	1	3	22	0	1
Yeh CH [13] 2020	38	137	6	17	36	133	10	19
Ohno Y [14] 2020	11	90	1	2	11	89	2	2
Ohno Y [15] 2021	16	71	6	5	21	67	2	8

*Abbreviations*: *TP* True positives, *TN* True negative, *FP* False positive, *FN* False negative

### Results of the risk-of-bias assessment

Table 3 summarizes the results of the risk-of-bias assessment using the QUADAS-2 tool. The risk of bias and applicability concerns regarding patient selection, which was interpreted as continuously enrolled, was unclear in 3 studies [3, 4, 7]. The risk of bias concerning the reference standard was high in all the studies because the reference standard results were confirmed by pathology or imaging follow-up data. Two studies [12, 13] were considered to have a high risk of bias with respect to applicability concerns of the reference standard because only follow-up imaging was used as the reference standard.

**Table 3:** Results of the quality assessment using the QUADAS-2 tool

Study,year	Risk of bias				Applicability concerns		
	Patient selection	Index test	Reference standard	Flow and timing	Patient selection	Index test	Reference standard
Ohno Y [2], 2015	Low risk	Low risk	High risk	Low risk	Low risk	Low risk	Low risk
Heusch P [3], 2015	Unclear	Low risk	High risk	Low risk	Unclear	Low risk	Low risk
Lee SM [4], 2016	Unclear	Low risk	High risk	Low risk	Unclear	Low risk	Low risk
Melsaether AN [5], 2016	Low risk	Low risk	High risk	Low risk	Low risk	Low risk	Low risk
Huellner MW [6], 2016	Low risk	Low risk	High risk	Low risk	Low risk	Low risk	Low risk
Sekine T [7], 2017	Unclear	Low risk	High risk	Low risk	Unclear	Low risk	Low risk
Catalano OA [8] 2017	Low risk	Low risk	High risk	Low risk	Low risk	Low risk	Low risk
Ohno Y [9] 2017	Low risk	Low risk	High risk	Low risk	Low risk	Low risk	Low risk
Botsikas D [10] 2019	Low risk	Low risk	High risk	Low risk	Low risk	Low risk	Low risk
Ohno Y [11] 2019	Low risk	Low risk	High risk	Low risk	Low risk	Low risk	Low risk
Liu Y [12] 2019	Low risk	Low risk	High risk	Low risk	Low risk	Low risk	High risk
Yeh CH [13] 2020	Low risk	Low risk	High risk	Low risk	Low risk	Low risk	High risk
Ohno Y [14] 2020	Low risk	Low risk	High risk	Low risk	Low risk	Low risk	Low risk
Ohno Y [15] 2021	Low risk	Low risk	High risk	Low risk	Low risk	Low risk	Low risk

### Results of the diagnostic accuracy of 18F-FDG PET/MRI

**Fig. 2:**
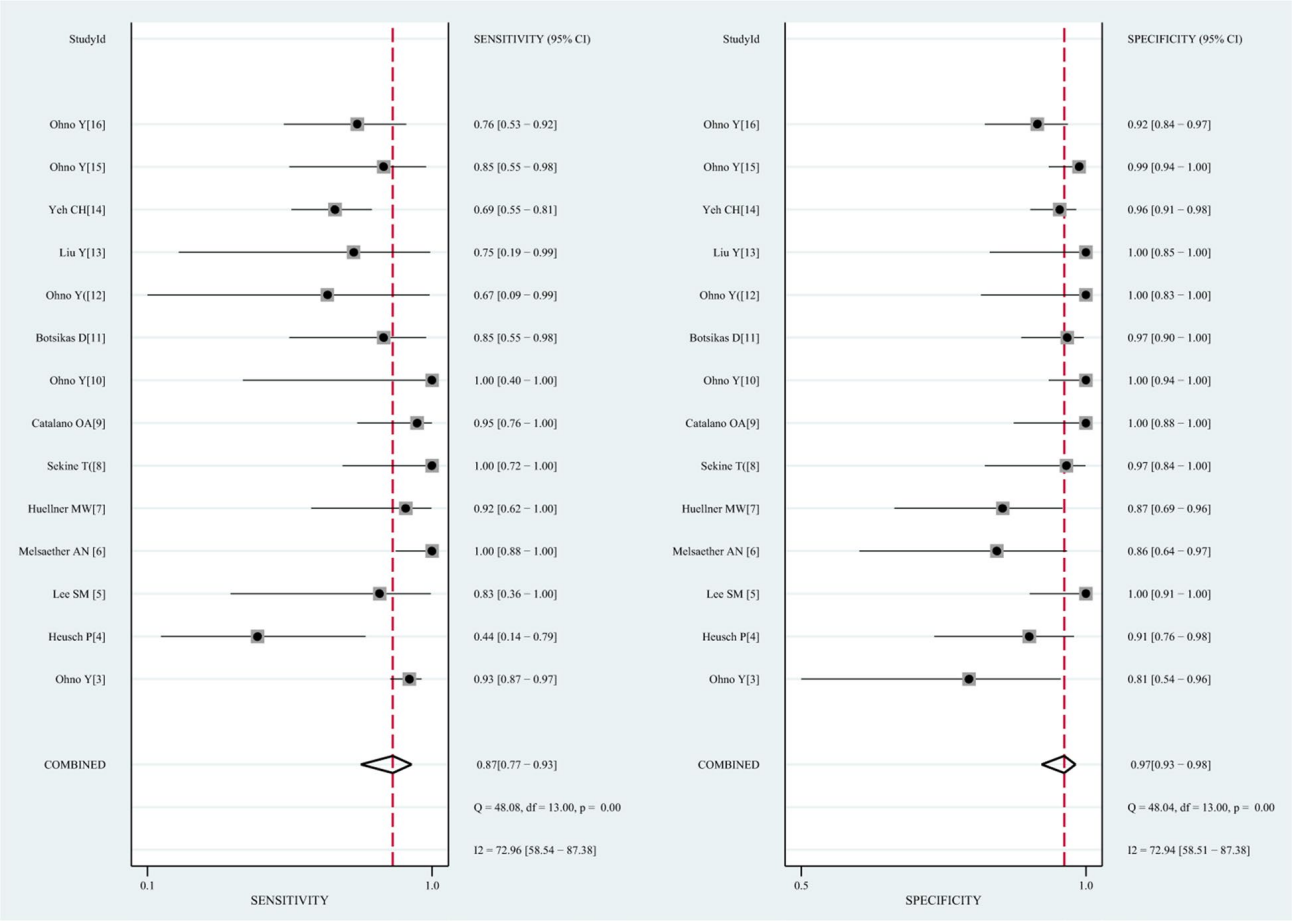
The forest plot of sensitivity and specificity of 18F-FDG PET/MRI for distant metastases in patients with malignant tumors

**Fig. 3:**
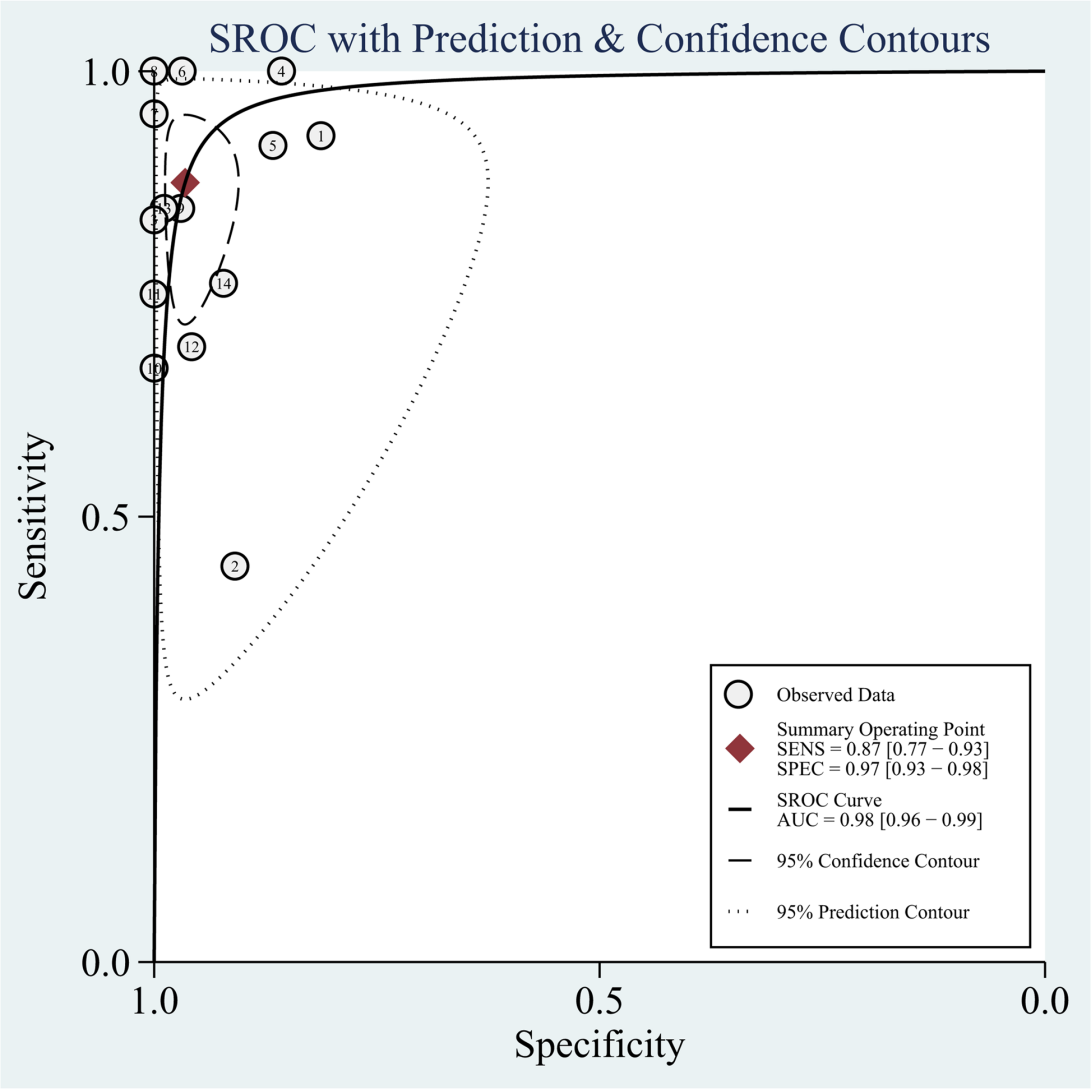
The forest plot of SROC curves of 18F-FDG PET/MRI for distant metastases in patients with malignant tumors

This study confirmed that significant heterogeneity existed in 18F-FDG PET/MR groups (*I*^2^ = 78%; *P* < 0.005), due to the threshold effect (Spearman correlation coefficient was -0.07, *P* = 0.01). In the meta-analytic summary, the pooled sensitivity, specificity, PLR, and NLR for 18F-FDG PET/MRI were 0.87 (95% confidence interval [CI] = 0.77 to 0.93), 0.97 (95% CI = 0.93 to 0.98), 25.1 (95% CI = 12.1 to 52.1), and 0.13 (95% CI = 0.07 to 0.24), respectively (Fig. 2). The SROC curve was located near the ideal upper left corner, and the AUC value was 0.98 (Fig. 3).

### Results of the diagnostic accuracy of 18F-FDG PET/CT

**Fig. 4:**
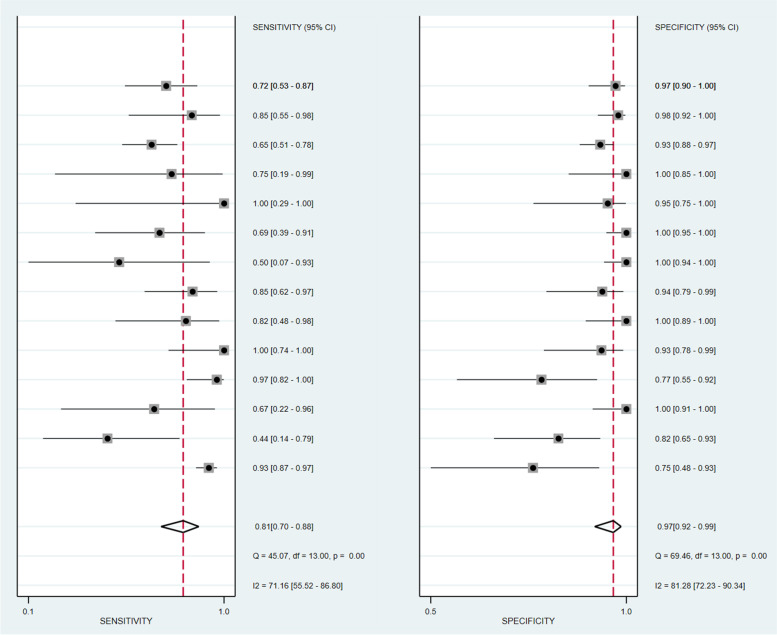
The forest plot of sensitivity and specificity of 18F-FDG PET/CT for distant metastases in patients with malignant tumors

**Fig. 5:**
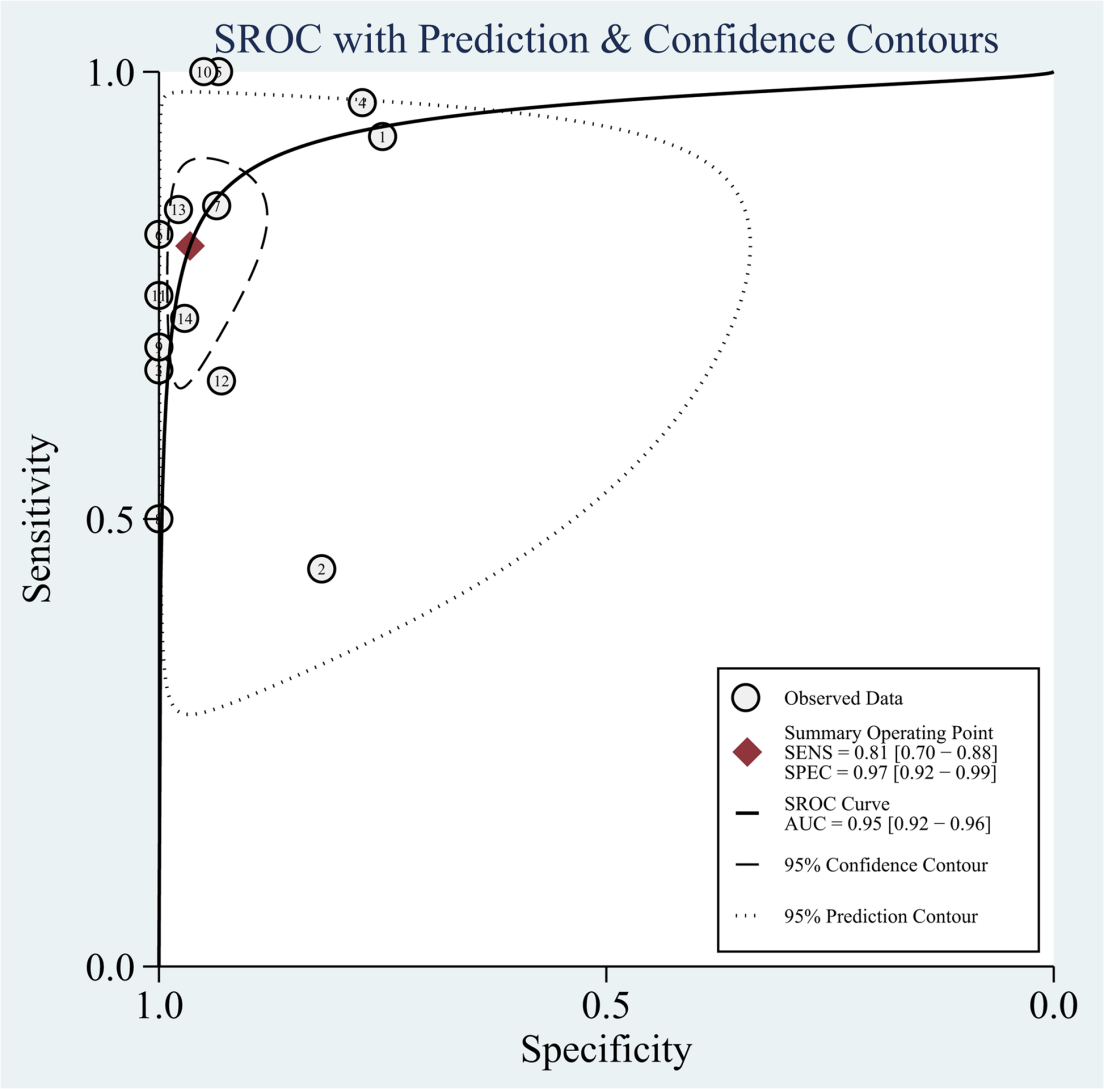
The forest plot of SROC curves of 18F-FDG PET/CT for distant metastases in patients with malignant tumors

This study confirmed that significant heterogeneity existed in 18F-FDG PET/CT groups (*I*^2^ = 91%; *P* < 0.01), there is not threshold effect (Spearman correlation coefficient was -0.52, *P* = 0.27). The pooled sensitivity, specificity, PLR, and NLR for 18F-FDG PET/CT were 0.81 (95% CI = 0.70 to 0.88), 0.97 (95% CI = 0.92 to 0.99), 23.1 (95% CI = 9.5 to 56.0), and 0.20 (95% CI = 0.13 to 0.32), respectively (Fig. 4). The SROC curve was located near the ideal upper left corner, and the AUC value was 0.95 (Fig. 5).

### Subgroup analysis

A total of 3 studies of patients with breast cancer (182 patients) who had undergone both 18F-FDG PET/MRI and PET/CT. The weighted overall estimates of sensitivity, specificity, PLR, NLR, and AUC value for 18F-FDG PET/MRI were 0.95 (95% CI = 0.87 to 0.99), 0.96 (95% CI = 0.90 to 0.97), 15.85 (95% CI = 4.15 to 60.56), 0.09 (95% CI = 0.03 to 0.24), and 0.98, respectively; the weighted overall estimates of sensitivity, specificity, PLR, NLR, and AUC value for 18F-FDG PET/CT were 0.87 (95% CI = 0.76 to 0.94), 0.94 (95% CI = 0.88 to 0.98), 11.14 (95% CI = 2.59 to 47.86), 0.18 (95% CI = 0.07 to 0.46), and 0.94, respectively (Table 4). This suggests that 18F-FDG PET/MRI had higher sensitivity and specificity for detecting distant metastases of breast cancer than PET/CT.

**Table 4:** Diagnostic accuracy of 18F-FDG PET/MRI and PET/CT from including studies

Imaging tool	Clinical settings	No. of studies (no. of patients)	Independent estimates		Likelihood ratio		AUC
			Sensitivity (95% CI)	Specificity (95% CI)	PLR (95% CI)	NLR (95% CI)	
PET/MRI	All studies	14(1042)	0.87(0.77–0.93)	0.97(0.93–0.98)	25.1(12.1–52.1)	0.13(0.07–0.24)	0.98
PET/CT	All studies	14(1042)	0.81(0.70–0.88)	0.97(0.92–0.99)	23.1(9.5–56.0)	0.20(0.13–0.32)	0.95
PET/MRI	Lung cancer	5(429)	0.84(0.71–0.92)	0.96(0.88–0.99)	21.8(6.9–68.7)	0.16(0.09–0.30)	0.95
PET/CT	Lung cancer	5(429)	0.87(0.77–0.93)	0.95(0.85–0.98)	16.6(5.6–49.2)	0.13(0.07–0.24)	0.94
PET/MRI	Breast cancer	3(182)	0.95(0.87–0.99)	0.96(0.90–0.97)	15.9(4.2–60.6)	0.09(0.03–0.24)	0.98
PET/CT	Breast cancer	3(182)	0.87 (0.76–0.94)	0.94 (0.88–0.98)	11.1 (2.6–47.9)	0.18(0.07–0.46)	0.94

*Abbreviations*: *CI* Confidence interval, *PLR* Positive likelihood ratio, *NLR* Negative likelihood ratio, *AUC* Area under summary receiver operating characteristic curve

A total of 5 studies of patients with lung cancer (429 patients) who had undergone both 18F-FDG PET/MRI and PET/CT. The weighted overall estimates of sensitivity, specificity, PLR, NLR, and AUC value for 18F-FDG PET/MRI were 0.84 (95% CI = 0.71 to 0.92), 0.96 (95% CI = 0.88 to 0.99), 13.8 (95% CI = 6.9 to 68.7), 0.16 (95% CI = 0.09 to 0.30), and 0.95, respectively; the weighted overall estimates of sensitivity, specificity, PLR, NLR, and AUC value for 18F-FDG PET/CT were 0.87 (95% CI = 0.77 to 0.93), 0.95 (95% CI = 0.85 to 0.98), 16.6 (95% CI = 5.6 to 49.2), 0.13 (95% CI = 0.07 to 0.24), and 0.94, respectively (Table 4). This suggests that 18F-FDG PET/CT had higher sensitivity for detecting distant metastases of lung cancer than PET/MRI.

### Analysis of publication bias

**Fig. 6:**
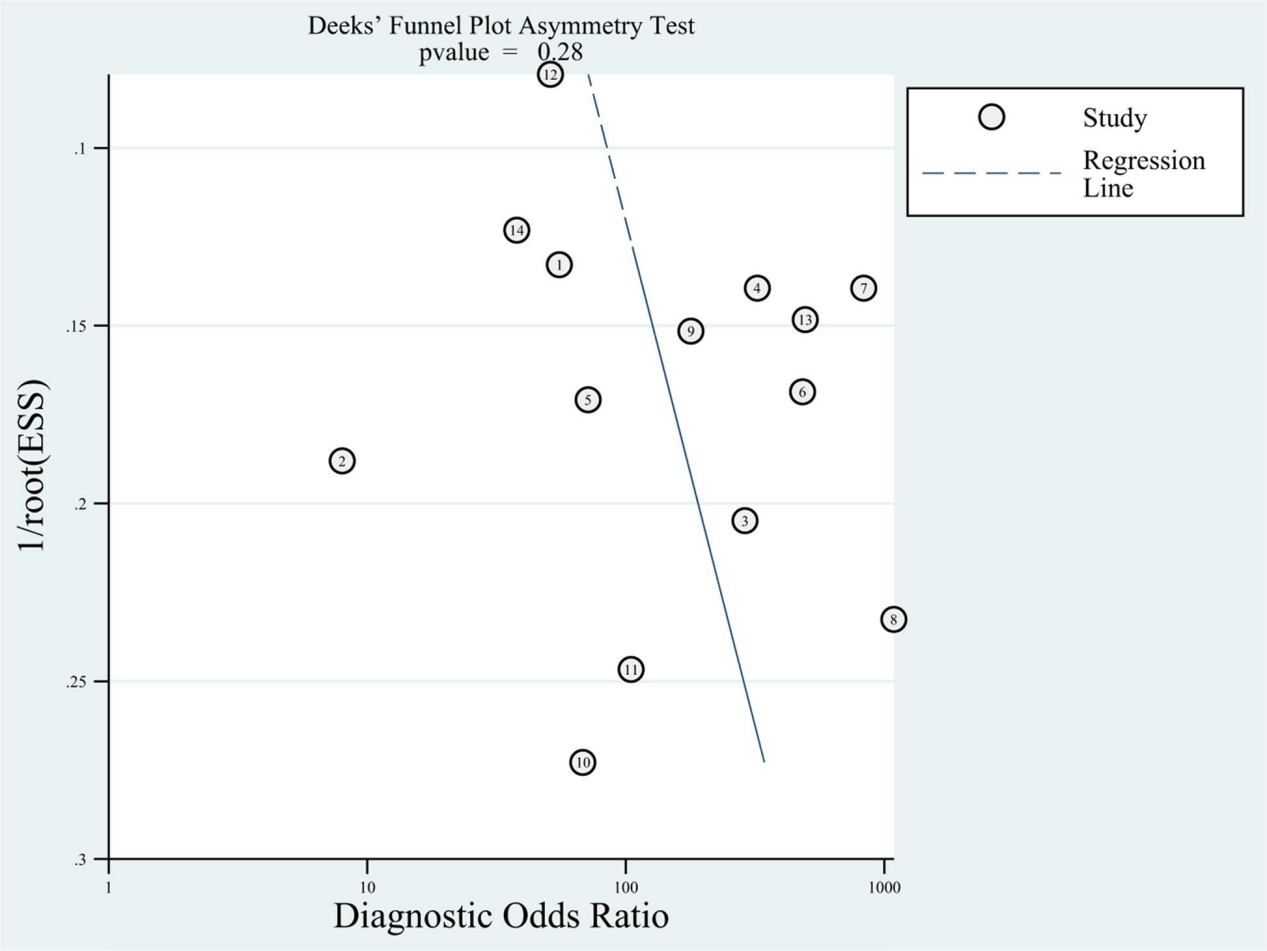
Deeks funnel plot of asymmetry test for publication bias of PET/MR

**Fig. 7:**
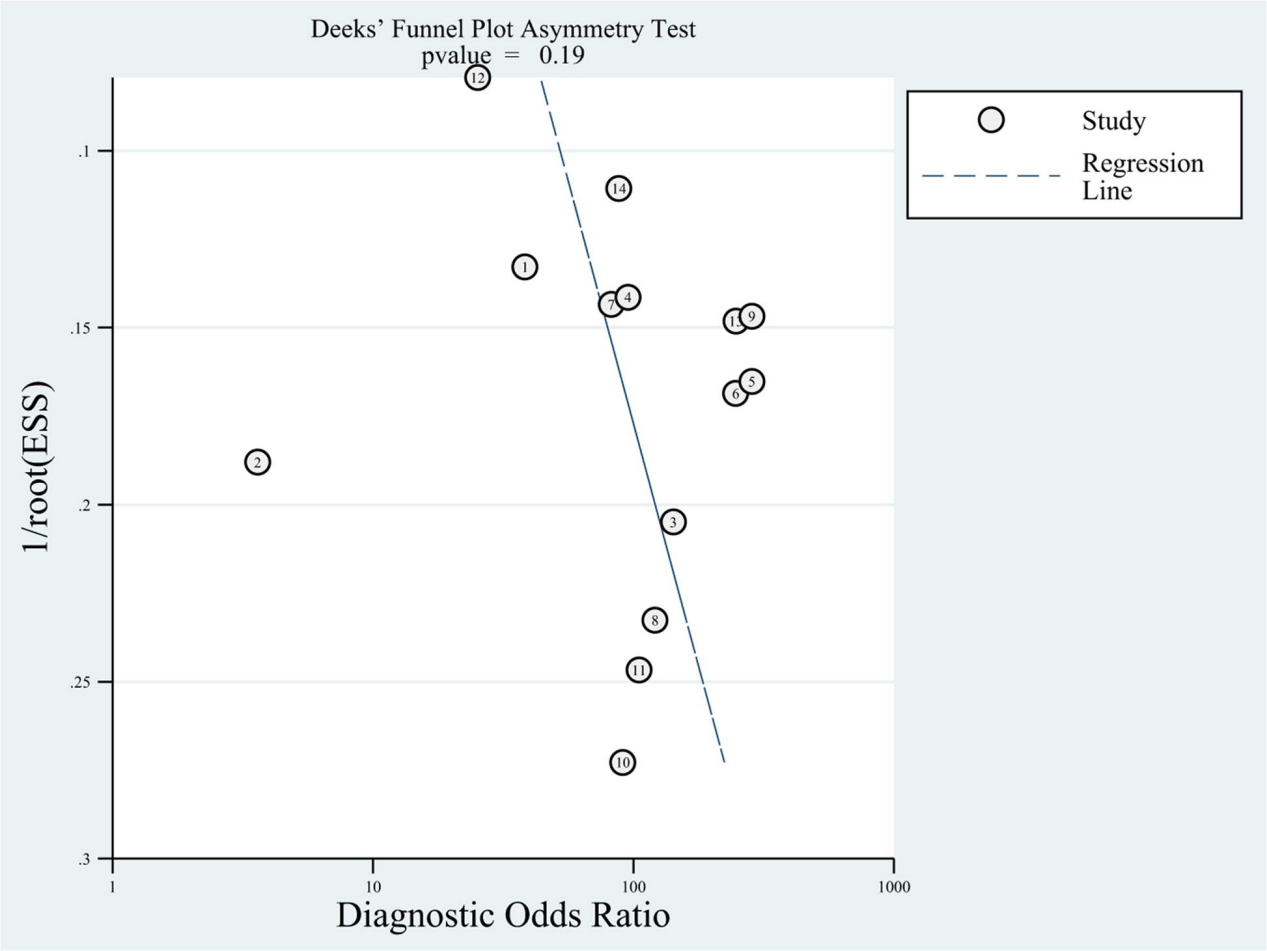
Deeks funnel plot of asymmetry test for publication bias of PET/CT

The results of Deek’s funnel plots were not significant for 18F-FDG PET/MR (*P* = 0.281 > 0.05, t = 1.13, 95% CI = -7.57 – 23.77) (Fig. 6) and for 18F-FDG PET/CT (*P* = 0.194 > 0.05, t = 1.38, 95% CI = -4.91 – 21.73) (Fig. 7), suggesting no major publication bias.

## Discussion

Despite improvements in treatment techniques, advanced cancer with distant metastases remains difficult to cure. When making decisions on advanced cancer therapy, it is necessary to have a precise assessment of possible distant metastases. With the development of imaging technology, the clinical application of whole-body 18F-FDG PET/MRI and PET/CT may make it possible for distant metastases to be effectively detected in patients with advanced cancer. The current meta-analysis demonstrated that 18F-FDG PET/MRI had a higher diagnostic accuracy for detecting distant metastases than PET/CT with a higher sensitivity (0.87 versus 0.81) and a higher AUC value (0.98 versus 0.95). Combining the evidence from the included studies in this meta-analysis, it can be concluded the following main reasons for that outcome.

The main reason for the higher sensitivity of 18F-FDG PET/MRI come from the MRI section of PET/MRI, MRI can provide additional enhancement properties, DWI, and relaxation time-dependent information such as signal intensity (SI) assessments. Ohno et al. [17] reported that the sensitivity of whole-body MRI with DWI for recurrence assessment of lung cancer was higher than that of whole-body MRI without DWI (88.2% versus 70.6%), indicating that the addition of DWI could improve the diagnostic accuracy of whole-body MRI. The diagnostic accuracy for M1 staging of malignant pleural mesothelioma of whole-body 18F-FDG PET/MRI with SI assessment (95.7%) was higher than that of PET/MRI without SI assessment (87.0%), indicating that SI assessment could provide additional information to improve the diagnostic accuracy of whole-body PET/MRI [11]. Moreover, Ohno Y [2] stated that whole-body PET/MRI with SI assessment can assist in accurately evaluating regional lymph node involvement, presence of distant metastatic specificity, and clinical stage in patients with lung cancer. Melsaether AN et al. [5] reported that 15 brain metastases in breast cancers were seen on contrast-enhanced T1-weights images of MRI, but no metabolic activity was measured in PET images and have resulted in FN results on PET/CT. The PET/MRI protocol of most studies included in this meta-analysis included gadolinium-containing contrast enhancement, DWI, and SI assessment, which might provide incremental value for the diagnosis of metastases.

Another important issue that attributed to improving the diagnostic performance of 18F-FDG PET/MRI is FDG avidity of PET data. 18F-FDG uptake affects diagnostic accuracy, on the one hand, non-FDG-avid lesions such as permeative osseous metastases and sub-centimeter hepatic, brain metastases are not visible on PET, but on MRI [8]. On the other hand, metastases lesions that are located in organs with high background FDG activity, such as the adrenal glands, which show variable physiologic FDG uptake, are missed on PET/CT and might result in FP, but are visible on MRI [18–20]. It has become clear that the pattern of 18F-FDG kinetics and 18F-FDG uptake varies by different histology types and histology grading, causing differences in diagnosis accuracy [21]. Among invasive breast carcinomas, 18F-FDG uptake in “carcinoma in situ” is usually weak [22], invasive carcinomas with high Scarff-Bloom-Richardson grade exhibit higher 18F-FDG uptake than carcinomas of lower grade, and invasive ductal carcinoma exhibits higher 18F-FDG uptake than invasive lobular carcinoma [23]. In our inclusion studies, its available data were obtained with mixed subtypes of breast cancers, which has hampered the accuracy assessment of subtype-specific of breast cancer with 18F-FDG PET/CT and PET/MRI.

Besides, it is worth highlighting that 18F-FDG PET/MRI and PET/CT have different advantages for detecting metastatic organs. On the one hand, 18F-FDG PET/MRI may be superior for detecting subcutaneous, brain, liver, and bone metastases due to its high soft-tissue contrast, such as lymph node metastases or bone metastases is easy to visualize on short inversion time inversion recovery sequence, adrenal gland metastasis can be depicted on dual-phase T1-gradient echo sequence, brain and liver metastases can be seen on contrast-enhanced T1-weighted images. Lee SM et al. found that one brain metastasis was missed on PET/CT, which depicted the weakness of PET/CT in detecting brain metastases [4]. Available data show that PET/CT depicts 50–70% of known presumably symptomatic brain metastases [24]. Botsikas D et al. [10] showed that PET/MRI had a significantly higher sensitivity than PET/CT for detecting bone metastases. The reason for this difference is that the T1-weighted imaging sequence reveals bone metastatic lesions by identifying bone marrow infiltration, but a faint radiotracer uptake on PET that is not associated with a corresponding CT finding results in a negative on PET/CT. Beiderwellen et al. [25] found that PET/MRI provided superior ability to display lesion for bone metastases and enabled the delineation of more malignant lesions than PET/CT. Melsaether AN et al. [5] found that PET/MRI outperformed PET/CT for detecting bone and liver metastases in a lesion-based analysis. On the other hand, 18F-FDG PET/CT can be expected to be advantageous in the detection of small pulmonary metastases as sub-centimeter pulmonary nodules were seen on PET/CT images, but PET/MRI was less sensitive for detecting small nodules because they are not FDG avid [26]. However, this study did not compare the differences in accuracy in determining the organ-specific metastases between 18F-FDG PET/CT and PET/MRI because the data regarding organ-specific metastases could not be extracted from those studies.

In the subgroup analysis, 18F-FDG PET/MRI had a higher sensitivity and specificity than PET/CT for evaluating distant metastases of breast cancers. This is in accordance with the findings of another systematic review conducted by de Mooij et al. [27], who also concluded that 18F-FDG PET/MRI has achieved higher diagnostic accuracy than 18F-FDG PET/CT in the distant staging of patients with breast cancer. Botsikas D et al. [10] compared the whole-body PET/MRI with PET/CT in breast cancer and found no statistically significant difference in the sensitivity, specificity, PPV, or NPV of the two tools for detecting distant metastases in a patient-per-patient analysis. In breast cancer, the bone is a common site for distant metastases and occurs in 69% of patients with advanced disease. Previous studies have found that PET/MRI detected osseous metastases in significantly more patients with breast cancer than PET/CT [28]. Melsaether AN et al. [5] reported that PET/MRI showed higher sensitivity than PET/CT for detecting liver metastases in breast cancers owing to DWI. It seems that PET/MRI has higher diagnostic confidence likely due to the superiority over detected lesions in bone, and liver metastases, compared with PET/CT.

In another subgroup analysis, 18F-FDG PET/CT had a higher sensitivity and a lower specificity for detecting distant metastases of lung cancer than PET/MRI. PET/MRI detected slightly more bone and liver metastases than PET/CT [29], however, the detection of FDG-avid nodules is poorer with PET/MRI than with PET/CT [30]. Despite all this, PET/MRI has been suggested to match or surpass the accuracy of PET/CT for the staging and recurrence surveillance of multiple thoracic malignancies [15].

Based on the results summarized in this meta-analysis, 18F-FDG PET/CT has been demonstrated to achieve similar diagnostic performance in the distant staging of breast and lung cancer (sensitivity 0.87 versus 0.87). 18F-FDG PET/MRI has higher accuracy in the distant staging of breast cancer to lung cancer (sensitivity 0.95 versus 0.84).

This present meta-analysis has some limitations that should be considered. First, inadequate data was acquired from the included studies to separately evaluate the diagnostic accuracy based on per-lesion analysis. Second, pathological examinations from biopsies to confirm metastatic lesions were not obtained from every metastatic lesion. The imaging follow-up also used as the reference standard when a pathological examination was missing. Third, there was considerable heterogeneity between the studies, although we performed subgroup analyses. Fourth, different MRI protocols were taken in different studies, there are no standard MR sequence protocols in the included studies, plus DWI and a dedicated MRI of the breast were used in some studies.

## Conclusion

This meta-analysis highlights that 18F-FDG PET/MRI and PET/CT both performed well as detectors of distant metastases in advanced patients with malignant tumors, and the former has higher sensitivity. The subgroup analysis highlights that 18F-FDG PET/MRI and PET/CT hold different advantages for distant metastases staging in the different tumors, PET/MRI has a higher accuracy in patients with breast cancer patients, while PET/CT has a higher accuracy in patients with lung cancer.

## Data Availability

All data generated or analyzed during this study are included in this published article.

## Abbreviations

CT: Computed tomography
MRI: Magnetic resonance imaging
PET/CT: Positron emission tomography/computed tomography
PET/MRI: Positron emission tomography/magnetic resonance imaging
CI: Confidence intervals
QUADAS: Quality Assessment of Diagnostic Accuracy
PLR: Positive likelihood ratio
NLR: Negative likelihood ratio
SROC: Summary receiver operating characteristic
